# Acute Myocardial Infarction After COVID-19 Vaccination: A Case Report

**DOI:** 10.7759/cureus.25536

**Published:** 2022-05-31

**Authors:** Animesh Mishra, Ojing Komut, Arun Kumar, Tony Ete, Rinchin D Megeji

**Affiliations:** 1 Cardiology, North Eastern Indira Gandhi Regional Institute of Health and Medical Sciences, Shillong, IND; 2 General Surgery, Tomo Riba Institute of Health and Medical Sciences, Naharlagun, IND; 3 Cardiology, North Eastern Indira Gandhi Regional Institute of Health And Medical Sciences, Shillong, IND; 4 Cardiology, Tomo Riba Institute of Health and Medical Sciences, Naharlagun, IND

**Keywords:** covid-19, primary coronary angioplasty, coronary artery angiography, st-elevation myocardial infarction (stemi), post-vaccination

## Abstract

Following the coronavirus disease 2019 (COVID-19) pandemic, nations all over the world started vaccination programs against the SARS-CoV2 virus. With the widespread administration of the vaccine across the globe, various cases were reported with thrombotic events after vaccination. Here, we are presenting a case of acute anterior wall myocardial infarction (AWMI) after ChAdOx1 nCoV- 19 corona virus (recombinant) vaccination. A 68-year-old male who was a known case of hypertension, non-smoker on antihypertensive took COVISHIELD vaccination and presented with acute anterior wall myocardial infarction within 12 hours and was taken up for primary angioplasty. On coronary angiography, mid-left anterior descending artery (LAD) was 99% stenosed. Following angiography percutaneous transluminal coronary angioplasty (PTCA), deployment of a drug eluting stent was done. Post-procedure time was uneventful. He was started on intravenous fluids and amiodarone infusion. The patient recovered and was discharged in stable condition. The leading approach to handling COVID-19 pandemic is mass vaccination. In this case, the MI after vaccination might be coincidental. We want to highlight this case as that the complication can occur during the mass vaccination programs and hence adequate precautionary measures like basic life support, EKG monitoring, and emergency ambulance services should be present in all primary and community health centers (PHC and CHC). This will help in avoiding the COVID vaccination hesitancy among the general public.

## Introduction

After the world was struck by the coronavirus disease 2019 (COVID-19) pandemic, nations across the globe started implementing various treatment guidelines and preventive measures for their population. One of the most important preventive measures in this pandemic is to provide vaccine against COVID-19. Out of 308 vaccine candidates which are in various stages of development, only 18 vaccines were approved by the world health regulatory agencies including two RNA vaccines (Pfizer-BioNTech and Moderna), nine conventional inactivated vaccines (BBIBP-CorV, Chinese Academy of Medical Sciences, CoronaVac, Covaxin, CoviVac, COVIran Barakat, Minhai-Kangtai, QazVac, and WIBP-CorV), five viral vector vaccines (Sputnik Light, Sputnik V, Oxford-AstraZeneca, Convidecia, and Johnson & Johnson), and two protein subunit vaccines (EpiVacCorona and RBD-Dimer). In India, there are three vaccines available for use. They are COVISHIELD (ChAdOx1 nCoV-19 adenovirus vaccine) - a brand of Oxford-AstraZeneca, COVAXIN (inactivated virus), and the SPUTNIK V (human adenovirus vaccine) with adequate safety and efficacy [1]. As the mass COVID-19 vaccination program started, there were some cases of blood clots and thromboembolic events among vaccinated individuals. This raised concerns among the world nations. In India and western countries, researchers reported many such cases of arterial and venous thrombosis after adenovirus-based COVID-19 vaccines [2-4]. Here, we are presenting a case of a 68-year-old male presented with chest pain diagnosed as acute ST elevation anterior wall myocardial infarction within 12 hours after taking the COVISHIELD vaccine.

## Case presentation

**Figure 1:**
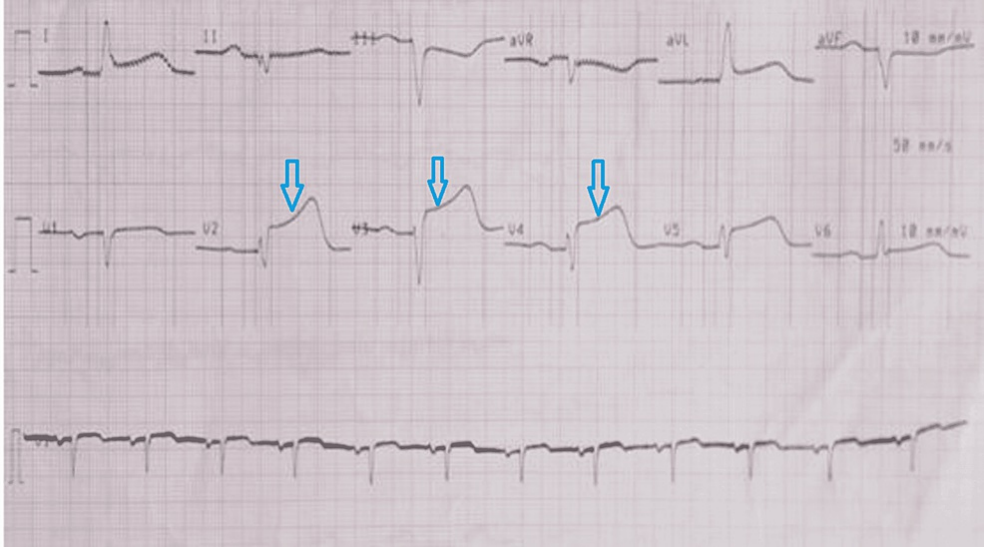
Electrocardiogram showing ST elevation in leads V2-V5

A 68-year-old male patient who is a non-smoker, non-alcoholic, and a known case of hypertension on regular medication, with no history suggestive of any allergy was administered the COVISHIED vaccine. On the day of vaccination, he went for a general check-up, his vitals were stable and his electrocardiogram was within normal limits. After a routine check-up, the patient received his first dose of COVISHIELD (ChAdOx1) - chimpanzee-based adenovirus vaccine. After vaccination, the patient was kept under observation for an hour. Following any uneventful episode, he was released after an hour of vaccination. Initially, he was comfortable at home but after 10-12 hours of vaccine administration, the patient developed typical anginal chest pain and was taken to the nearest hospital where a diagnosis of acute anterior wall myocardial infarction (AWMI) was made following changes in the electrocardiogram and elevated cardiac enzymes. ECG showed ST elevation in V2-5 (Figure 1).

**Figure 2:**
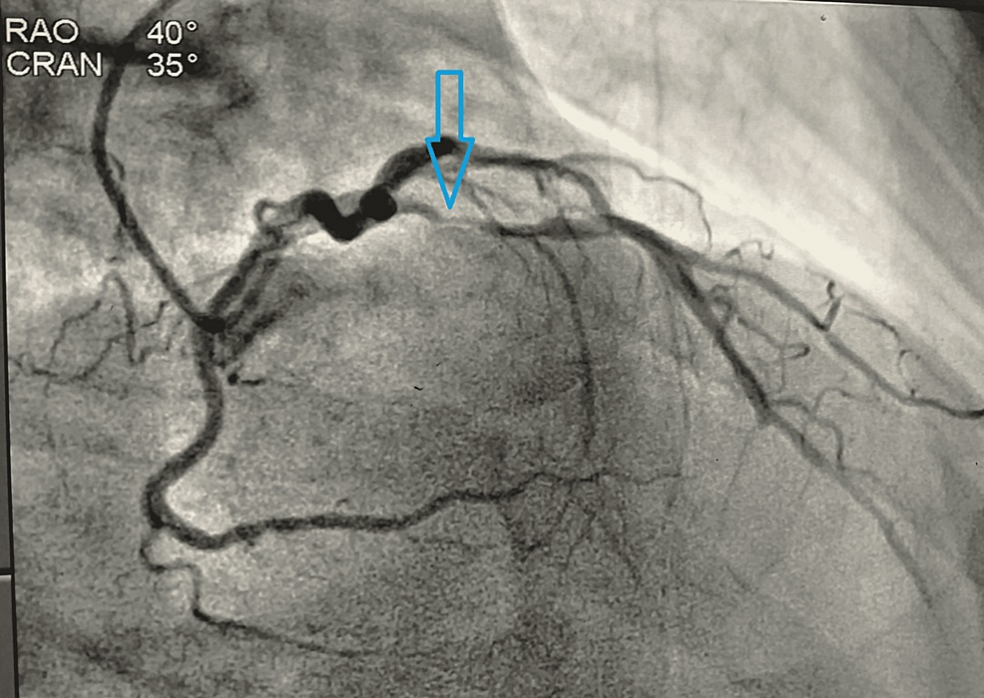
Coronary angiogram in RAO view showing stenosis (90-95%) of LAD artery RAO: right anterior oblique; LAD: left anterior descending

Echocardiography revealed hypokinesia in basal mid anterior and anteroseptal segments with an ejection fraction of 40%. Following this, he was treated with antiplatelets and statin and referred to our center which provides a tertiary level referral care. The cartridge-based nucleic acid test (CB-NAAT) for COVID-19 was negative. The patient was shifted to the cardiac catheterization laboratory for primary percutaneous coronary intervention (PCI) after informed consent. Coronary angiogram was done which revealed discrete significant stenosis around 90-95% of the left anterior descending artery (LAD) artery (Figure 2). 

Percutaneous transluminal coronary angioplasty (PTCA) of the culprit artery was carried out followed by deployment of drug eluting stent in LAD. Post-procedure was uneventful. After three days, the patient improved symptomatically and was discharged in stable condition. He is now on regular follow-up in our outpatient department (OPD).

## Discussion

There are very few case reports published in the literature on the association between COVID-19 vaccination and MI. In India, initially, there was some media coverage reporting COVID-19 vaccine-related deaths. The first case related to post-COVID 19 vaccination death was a 43-year-old worker, who died due to MI after three days of COVISHIELD vaccination. Another two were reported in 65-year-old and 75-year-old males, who also had pre-existing comorbidities. The local district and state health committee found that these comorbid conditions were the actual cause of death and concluded it to be coincidental to vaccination. Kumar et al. presented two case reports with the diagnosis of acute inferior wall myocardial infarction (IWMI) and acute AWMI after the COVISHIELD vaccine within 20 hours and 30 minutes, respectively [3]. In the United States, Boivin and Martin reported a 96-year-old female, a known hypertensive presented with acute IWMI within one hour after taking the MODERNA vaccine [4]. Chatterjee et al. reported a case of a 63-year-old male without any comorbid conditions who presented with acute IWMI after the third day of COVISHIELD vaccine [5]. Tajstra et al. reported a case of an 85-year-old male with a history of prostatic cancer treated with prostatectomy and radiotherapy, who was diagnosed with acute IWMI within 30 minutes of receiving the first dose of Pfizer-BioNTech vaccine [6]. Jones et al. reported a case of a 63-year-old male patient who presented with pulmonary thromboembolism and popliteal artery occlusion after receiving the first dose of ChAdOx1 nCoV-19 vaccine [7]. Greinacher et al. assessed the clinical and laboratory features of 11 patients in whom thrombosis or thrombocytopenia had developed after vaccination with ChAdOx1 nCov-19 [8]. They concluded that the antibodies against platelet factor 4 can result in immune thrombotic thrombocytopenia, which clinically mimics heparin-induced thrombocytopenia. Wolf et al. analyzed the association between thrombotic episodes and COVID-19 Vaccine AstraZeneca (COVISHIELD) exposure. They concluded that it may trigger the antiplatelet antibodies which can cause thrombocytopenia with venous thrombotic episodes [9]. Scully et al. reported that the 23 patients developed thrombotic events six to 24 days after receiving the first dose of AstraZeneca (COVISHIELD) vaccine [10].

The above studies suggested that the antibodies to platelet factor 4 were related to thrombosis. Boivin and Martin stated that the post-vaccinated elderly individuals had myocardial supply demand mismatch which might be the aggravating factor of sudden cardiac arrest [4]. Genetic vaccines like mRNA vaccines (eg., Pfizer and Moderna) can directly infect platelets, mRNA translation, and spike protein synthesis. The viral vector-based vaccines (COVISHIELD and Sputnik V) can infect the megakaryocytes (the platelet precursor) which might be causing severe thrombocytopenia [11]. The other possible explanation could be an allergic reaction to vaccine causing vasospasic myocardial infarction termed as Kounis syndrome [12]. The severity of COVID-19 infection is high in those patients with comorbid conditions [13]; similarly, the risk of cardiac involvement is increased in COVID-19-vaccinated elderly individuals. Vaccination can induce inflammatory and immunological responses leading to prothrombotic state and increasing the demand for frail hearts in elderly individuals [4]. A health advisory has also been issued by the government of India regarding the very rare side effect of prothrombotic events after COVISHIELD vaccination. The European medical agency concluded that the Oxford-AstraZeneca COVID-19 vaccine is not linked to an increased risk of blood clots and is both safe and effective for vaccination. The benefit of vaccination outweighs the risk. At present, we don’t have any direct scientific evidence to prove any association between COVID-19 vaccine and thrombosis leading to MI. However, it is pertinent to raise this issue of an event like MI, whether causal or casual to avoid more complications and any loss of life.

## Conclusions

An association between two variables where a change in one makes a change in the other one is a causal association in epidemiology. In this case, other than an elderly age and hypertension, no other variables were present. However, it is also pertinent to mention here that increased age and hypertension itself increase the risk of cardiovascular events. Even though it is difficult to establish a causal relationship with a single case report but it is also important to highlight this complication of MI during vaccination against COVID-19, as it can occur during mass vaccination programs, especially in elderly individuals with comorbid conditions. In India, vaccination programs are mainly implemented through community and primary health centers. During vaccination programs, as a precautionary measure, these centers should have the basic ECG machines and life support facilities for the diagnosis of any event like MI. This will be helpful in prompt referral of these patients to tertiary cardiac care centers. Such an initiative will ensure public safety, the vaccine hesitancy among the general public, and in turn, increase the efficiency of vaccination programs. Also, most importantly the benefit of vaccination despite these rare events outweighs the risk of the COVID-19 disease.

## References

[1] Mandal S, Arinaminpathy N, Bhargava B, Panda S (2021). Responsive and agile vaccination strategies against COVID-19 in India. Lancet Glob Health.

[2] (2021). Two cardiac patients die after Covid shots. https://indianexpress.com/article/cities/kolkata/two-cardiac-patients-die-after-covid-shots/.

[3] Kumar B, Sabbarwal V, Nigam A, Gore P, Chauhan G, Darbari A (2021). Two case reports of acute ST-elevation myocardial infarction after COVID-19 vaccination: co-incidence or causal-association?. J Health Soc Sci.

[4] Boivin Z, Martin J (2021). Untimely myocardial infarction or COVID-19 vaccine side effect. Cureus.

[5] Chatterjee S, Ojha UK, Vardhan B, Tiwari A (2021). Myocardial infarction after COVID-19 vaccination-casual or causal?. Diabetes Metab Syndr.

[6] Tajstra M, Jaroszewicz J, Gąsior M (2021). Acute coronary tree thrombosis after vaccination for COVID-19. JACC Cardiovasc Interv.

[7] Jones M, Boisvert A, Landry J, Petrasek PF (2021). Limb ischemia and pulmonary artery thrombosis after the ChAdOx1 nCoV-19 (Oxford-AstraZeneca) vaccine: a case of vaccine-induced immune thrombotic thrombocytopenia. CMAJ.

[8] Greinacher A, Thiele T, Warkentin TE, Weisser K, Kyrle PA, Eichinger S (2021). Thrombotic thrombocytopenia after ChAdOx1 nCov-19 vaccination. N Engl J Med.

[9] Wolf ME, Luz B, Niehaus L, Bhogal P, Bäzner H, Henkes H (2021). Thrombocytopenia and intracranial venous sinus thrombosis after “COVID-19 vaccine AstraZeneca” exposure. J Clin Med.

[10] Scully M, Singh D, Lown R (2021). Pathologic antibodies to platelet factor 4 after ChAdOx1 nCoV-19 vaccination. N Engl J Med.

[11] Merchant HA (2021). CoViD vaccines and thrombotic events: possibility of mRNA translation and spike protein synthesis by platelets?. BMJ.

[12] Kounis NG, Mazarakis A, Tsigkas G, Giannopoulos S, Goudevenos J (2011). Kounis syndrome: a new twist on an old disease. Future Cardiol.

[13] Huang C, Wang Y, Li X (2020). Clinical features of patients infected with 2019 novel coronavirus in Wuhan, China. Lancet.

